# Tropomyosin diffusion over actin subunits facilitates thin filament assembly

**DOI:** 10.1063/1.4940223

**Published:** 2016-01-14

**Authors:** Stefan Fischer, Michael J. Rynkiewicz, Jeffrey R. Moore, William Lehman

**Affiliations:** 1 Computational Biochemistry Group, Interdisciplinary Center for Scientific Computing (IWR), University of Heidelberg, Im Neuenheimer Feld 368, D69120 Heidelberg, Germany; 2 Department of Physiology and Biophysics, Boston University School of Medicine, 72 East Concord Street, Boston, Massachusetts 02118, USA; 3 Department of Biological Sciences, University of Massachusetts Lowell, One University Avenue, Lowell, Massachusetts 01854, USA

## Abstract

Coiled-coil tropomyosin binds to consecutive actin-subunits along actin-containing thin filaments. Tropomyosin molecules then polymerize head-to-tail to form cables that wrap helically around the filaments. Little is known about the assembly process that leads to continuous, gap-free tropomyosin cable formation. We propose that tropomyosin molecules diffuse over the actin-filament surface to connect head-to-tail to partners. This possibility is likely because (1) tropomyosin hovers loosely over the actin-filament, thus binding weakly to F-actin and (2) low energy-barriers provide tropomyosin freedom for 1D axial translation on F-actin. We consider that these unique features of the actin-tropomyosin interaction are the basis of tropomyosin cable formation.

## INTRODUCTION

### Background

Tropomyosin, the first actin-binding protein to be identified (Bailey, 1946), was proposed by Crick (1953) to form a two-chained α-helical coiled-coiled, a structure then characterized further by Szent-Györgyi and Cohen (1957) and Holmes and Cohen (1963). A parade of elegant structural studies next revealed that tropomyosin molecules polymerize head-to-tail once bound to actin to form a continuous super-helical cable that wraps symmetrically around actin filaments (Hanson and Lowy, 1964; Spudich *et al.*, 1972; Flicker *et al.*, 1982; Phillips *et al.*, 1986; Milligan *et al.*, 1990; Vibert *et al.*, 1993; Lehman *et al.*, 1994; Greenfield *et al.*, 2006; and Hitchcock-DeGregori, 2008). The association of the coiled-coil cable on actin and its azimuthal shifting movements around the actin surface had already been suggested by Hanson and Lowy (1964) and by Huxley (1970, 1972) and later by others (Haselgrove *et al.*, 1972 and Parry and Squire, 1973) to sterically regulate the binding of myosin cross-bridges on actin and hence to control muscle contraction. In their review discussing how the structure of the tropomyosin “illuminates function,” Brown and Cohen (2005) pointed out that “tropomyosin is a… deceptively ‘simple’ molecule, designed, as it were, to interact with and match the actin helix.” Nevertheless, despite some 70 years of investigation, the structural mechanics of the tropomyosin molecule and its assembly onto the actin helix continue to be debated. The following commentary relates our perspective about the way that the structural blueprint of tropomyosin defines thin filament assembly, which then translates into muscle regulatory function.

### Single tropomyosin molecules and short oligomers of tropomyosin bind weakly to F-actin

True to the maxim that one's first thoughts can be one's worst thoughts, the binding properties of single tropomyosin molecules to actin filaments seem incompatible with thin filament assembly and function. In fact, individual tropomyosin molecules bind to actin with extremely low affinity (K_a_ ∼ 2 × 10^3^ M^−1^) (Wegner, 1980), equating to a few times kT; how then does tropomyosin bind to and regulate F-actin at all? Nevertheless, in what may seem to be improbable, equilibrium binding experiments show that F-actin, once saturated with tropomyosin, now interacts with the protein about 1000 times more strongly (*ibid.*), and hence, tropomyosin does remain bound to the assembled thin filament. Of course, nearest neighbor interaction dynamics account for this change in the apparent affinity. Namely, as tropomyosin begins to populate the actin filament, it forms head-to-tail connections, and as a consequence functions as a continuous cable on the actin substrate. Here, the binding is determined by the collective associations of multimerized tropomyosin. While the individual contributions of each tropomyosin-actin interaction may be weak and readily broken, the combined strength of the string of linked tropomyosin molecules wrapped around the actin filament prevents single tropomyosin molecules from diffusing away. Thus, transiently dissociating component molecules within the multimeric cable are likely to have their binding restored back onto the filament. In the case of the assembled thin filament, each individual tropomyosin molecule itself will only interact weakly with the underlying actin substrate. It therefore follows that at a local level, the weak interaction of individual tropomyosin molecules with actin still allows movement between regulatory positions that is essential to control muscle contraction at low energy cost. For example, tropomyosin position on the thin filament is readily perturbed by either troponin or myosin during muscle activation and relaxation. However, at a more global level, the continuity of the tropomyosin cable ensures that tropomyosin molecules collectively remain strongly bound to the thin filament, just as a rope wrapped snuggly around a tube will not dissociate even if the center does not hold. Nonetheless, this same rope might easily be pushed sideways.

### Electrostatic interaction maps reveal two distinct minima for tropomyosin position on F-actin

**FIG. 1.**
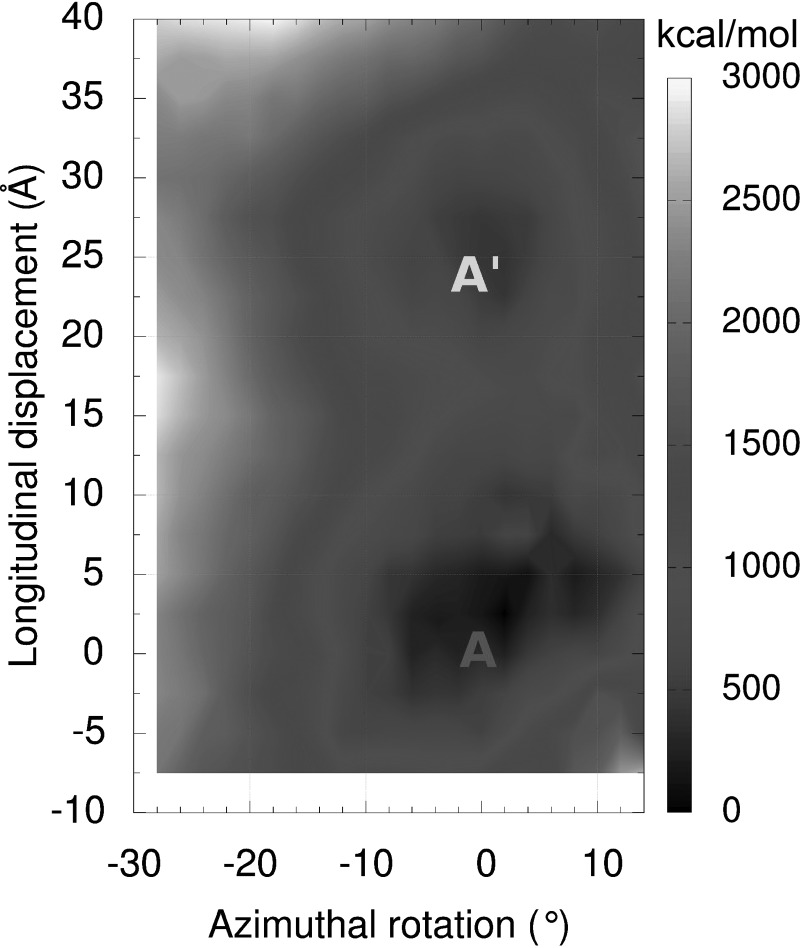
Coulombic interaction energy landscape of actin-tropomyosin. Each point in the energy landscape provides the electrostatic energy of interaction between F-actin and one tropomyosin coiled-coil, which has been repositioned longitudinally and azimuthally over F-actin (Rynkiewicz *et al.*, 2015). Positions at the two energy minima on the landscape are labeled A and A*′* and define models A and A′ structures shown in Figures 2 and 4. The energies (in kcal/mol) are given relative to the minimum energy position. Adapted from Rynkiewicz *et al.* (2015).

**FIG. 2.**
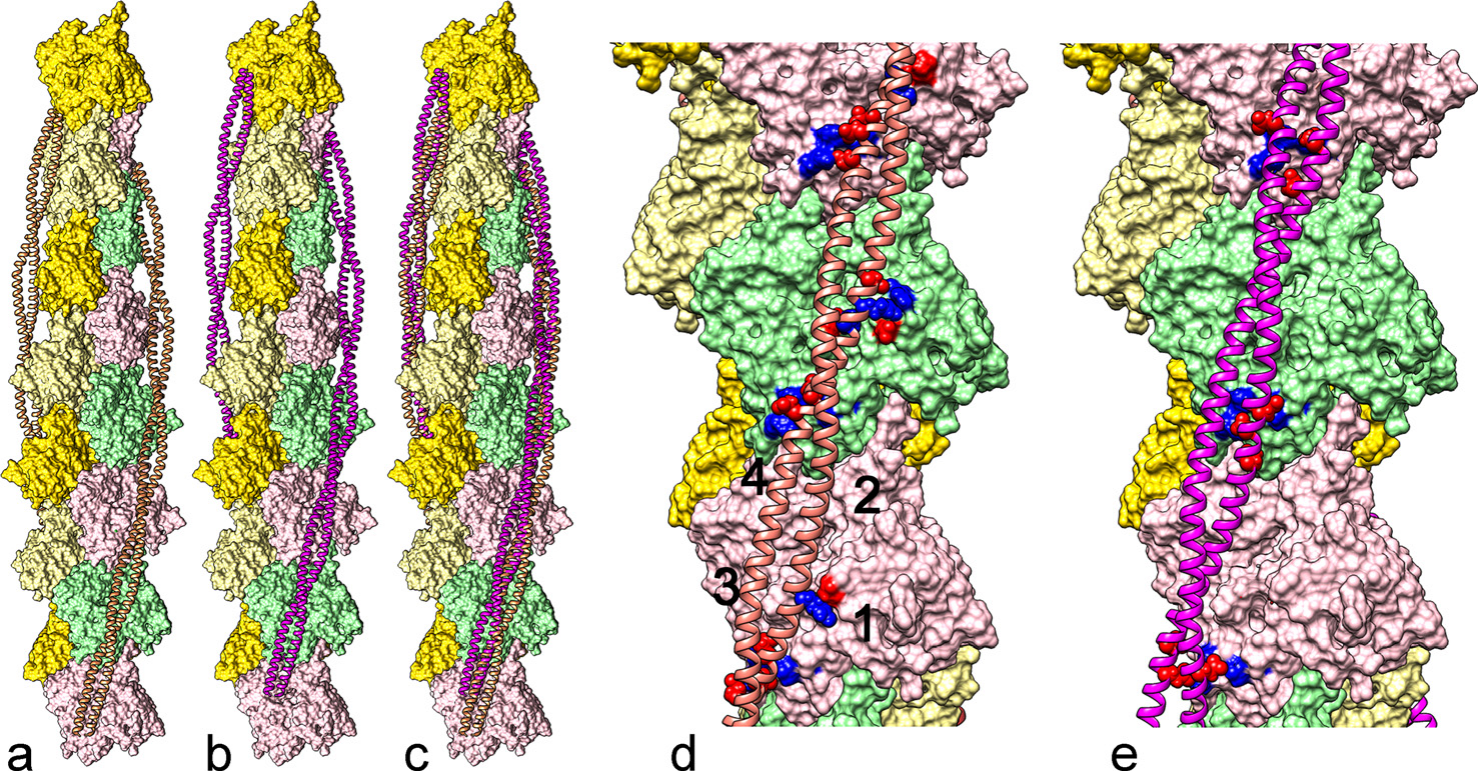
Tropomyosin positions on F-actin corresponding to the two energy minima. (a) The F-actin-tropomyosin model of Li *et al.* (2011) (Model A), matched to structures defined by the primary energy minimum in Figure 1 (tropomyosin, tan ribbons; actin, space filling models), (b) tropomyosin (magenta) translated axially by 24 Å over F-actin from its position in (a), producing a model which now fits to densities in cryo-EM reconstructions (von der Ecken *et al.*, 2014 and Rynkiewicz *et al.*, 2015), while matching structures defined by the second energy minimum in Figure 1 (model A′), (c) panels (a) and (b) superimposed, (d) and (e) enlargements of (a) and (b) highlighting charged amino acids on actin subunits largely responsible for electrostatic interactions with tropomyosin (Arg147, Lys326, Lys 328, Arg28 (each blue), and Asp25 (red)). Note that the model A′ defined by the second minimum does not contact the latter two residues on actin. Actin subdomains indicated on one actin subunit in (d). Of course, a transition between model A and model A′ under strain could account for muscle stretch-activation (cf. Perz-Edwards *et al.*, 2011). Models drawn using pdb coordinates from Orzechowski *et al.* (2014).

Interaction maps calculated between single tropomyosin molecules and the F-actin filament surface show two energy minima, each centered over a distinct but relatively shallow energy basin (Fig. 1; Rynkiewicz *et al.*, 2015). The two energy minima define two atomic models of the thin filament in which tropomyosin is translated longitudinally by 24 Å up and down F-actin and parallel to the filament axis (Fig. 2). This can be accomplished with minimal azimuthal rotation (Rynkiewicz *et al.*, 2015). The two structures fit uniquely and equally well to distinct EM-reconstructions of actin-tropomyosin preserved under different conditions (negative staining *vs*. rapid freezing) (Li *et al.*, 2011; von der Ecken *et al.*, 2014; and Rynkiewicz *et al.*, 2015). These results suggest that, as tropomyosin begins to assemble on F-actin, experimental conditions may bias weakly bound tropomyosin to occupy one or the other position on the filament depending on chemical milieu or filament strain. Given low energy barriers between the two energy minima on F-actin, it follows that, once bound, single tropomyosin molecules or short tropomyosin oligomers can translate between corresponding positions on the filament if unconstrained by actin-binding proteins. Although axial motion of a completed cable would involve breaking and reforming multiple, albeit weak interactions, short chains containing a few tropomyosin molecules might be able to diffuse axially, “skipping” between weakly associated interaction sites, and then coalesce into a longer chain at its lowest energy position. Such diffusion of tropomyosin over an F-actin substrate could be considered analogous to the well-known one-dimensional diffusion displayed by DNA-binding proteins linked to DNA as they search for and target specific recognition sites on a DNA polymer (reviewed in Halford and Marko, 2004 and Bauer and Metzler, 2012).

As mentioned, the two atomic models of the actin-tropomyosin filament are defined by distinct energy minima which differ from each other by a pure translation of tropomyosin parallel to the filament longitudinal axis. Therefore, interactions between tropomyosin and F-actin subunits described by the model for the primary energy minimum, with the deepest energy well, will necessarily differ from those predicted by the model representing the second minimum. The residue-residue specificity between tropomyosin and F-actin defined by the first model has been previously noted by several groups (Brown and Cohen, 2005; Li *et al.*, 2011; and Barua *et al.*, 2013) (Figs. 2(a) and 2(d)). The second model recently proposed by Rynkiewicz *et al.* (2015) (Figs. 2(b) and 2(e)) is consistent with newly formulated cryo-EM data (von der Ecken *et al.*, 2014 and Rynkiewicz *et al.*, 2015). Taken together, the two models prompt reinterpretation of the significance of tropomyosin sequence periodicity so elegantly outlined by McLachlan and Stewart (1976).

## RESULTS, ANALYSIS, AND DISCUSSION

**FIG. 3.**
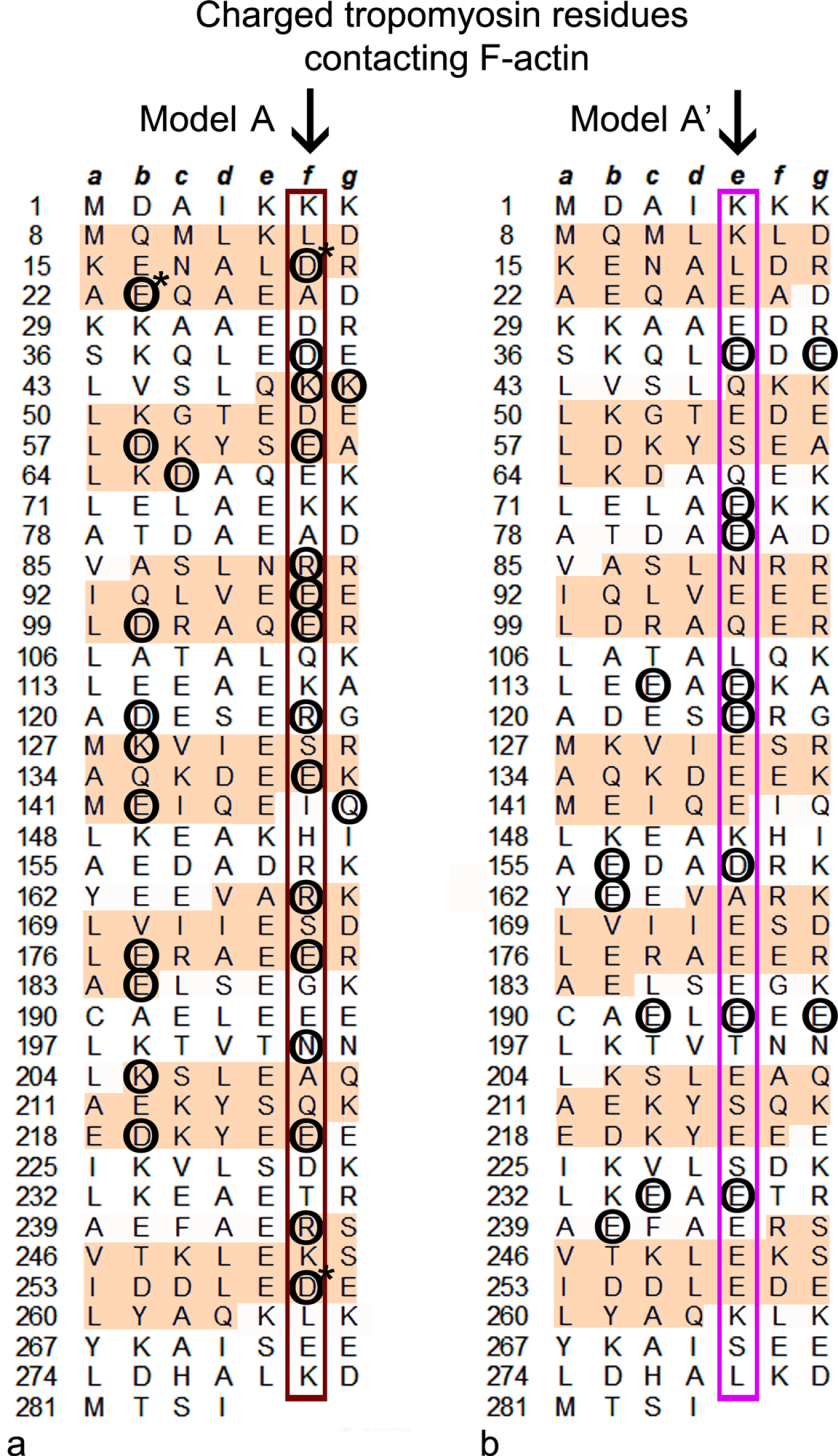
Striated muscle α-tropomyosin sequence annotation specifying charged amino acids in model A and model A′ that contact actin. Figure format based on Brown *et al.* (2001) to highlight tropomyosin heptad repeats (*a-g*) with the beginning residue of each repeat numbered. Alternating α- and β-zone sequences are differentiated by shading the α-zones tan. (a) and (b) Charged residues on tropomyosin that that lie within 5 Å of oppositely charged residues on actin in either of the two models are circled (based on data from Rynkiewicz *et al.*, 2015). Circled residues marked with an asterisk based on data from Li *et al.* (2011) and Orzechowski *et al.* (2014). (a) Note residues occupying *f-*positions in model A show a high frequency of such interactions, while in (b) those in *e*-positions in model A′ now show a predilection for such interactions (indicated in each case by arrows and rectangular bordering).

McLachlan and Stewart (1976) noted that the 284 residue long tropomyosin sequence can be divided into a repeating pattern of seven sets of tandem “α”- and “β”-bands, each composed of roughly 19 to 20 residues. Taken together, α- and β-band sets represent tropomyosin “pseudo-repeats” (39–40 residues) designed to target successive actin subunits along the thin filament (Fig. 3). Phillips *et al.* (1986) observed that α-band sequences are far more regular than the ones in the β-band arrays, as can be seen in Figure 3. Phillips and colleagues, therefore, concluded that α-bands are more likely to be responsible for F-actin binding, which is completely consistent with current models of the thin filament (Brown and Cohen, 2005; Li *et al.*, 2011; and Barua *et al.*, 2013). In fact, the structure shown in Figures 2(a) and 2(d) defined by the primary energy minimum noted above involves α-band residues of tropomyosin making electrostatic contacts with actin subdomains 1 and 3 on F-actin (Figs. 2 and 3(a)). Here, β-band residues lie largely over the shallow aspect of subdomains 2 and 4, at a radius too far from the actin surface to make meaningful contacts (Li *et al.*, 2011).

McLachlan and Stewart (1976) pictured tropomyosin undergoing a ∼90° “quarter-turn roll” during regulatory movement between relaxed and activated thin filament states so that in one case α-bands and in the other β-bands target complementary sites on actin. A rolling action of tropomyosin cable is now generally discounted (Lorenz *et al.*, 1995 and Rynkiewicz *et al.*, 2015), since it would require a large-scale unraveling of the tropomyosin cable on F-actin. It would also entail a high degree of torsional plasticity for tropomyosin to realize the coiled-coil pseudo-rotation accompanying the rolling, which appears to be inconsistent with measured tropomyosin mechanical properties (Li *et al.*, 2010a; Sousa *et al.*, 2010). However, a 24 Å longitudinal shifting or diffusion of tropomyosin, amounting to about the length of one-half an actin subunit, would not require tropomyosin cable unraveling or large-scale conformational plasticity (Figs. 2(a)–2(c)). However, a longitudinal shift would displace α-bands from their interaction sites on actin subunits, while now targeting β-bands to these same sites. This type of tropomyosin translation, in fact, produces an F-actin-tropomyosin structure shown in Figures 2(b) and 2(e), corresponding to the second energy minimum (Fig. 1) (Rynkiewicz *et al.*, 2015).

### Bending of tropomyosin to the contours of F-actin

Holmes and Lehman (2008) argued that actin filaments bind curved tropomyosin effectively because the coiled-coil protein is “preshaped” to the contours of the F-actin helix. Indeed, by following tropomyosin during molecular dynamics simulations, Li *et al.* (2010a; 2010b; 2010c) showed that the molecule's average shape matched the actin filament helix with remarkable fidelity. Inspection of the contours of crystal structures of full-length and shorter fragments of tropomyosin in fact had previously led Holmes to realize that innate helical curvature of the tropomyosin molecule parallels the actin helix quite closely (Li *et al.*, 2010a; also see Lehman *et al.*, 2014 and Rynkiewicz *et al.*, 2015). At first glance, an innate tropomyosin curvature may seem inconsistent with the existence of two unique binding sites on actin. However, the “deceptively simple” tropomyosin molecule structure can account for two binding mode sets between the canonical coiled-coil and actin.

**FIG. 4.**
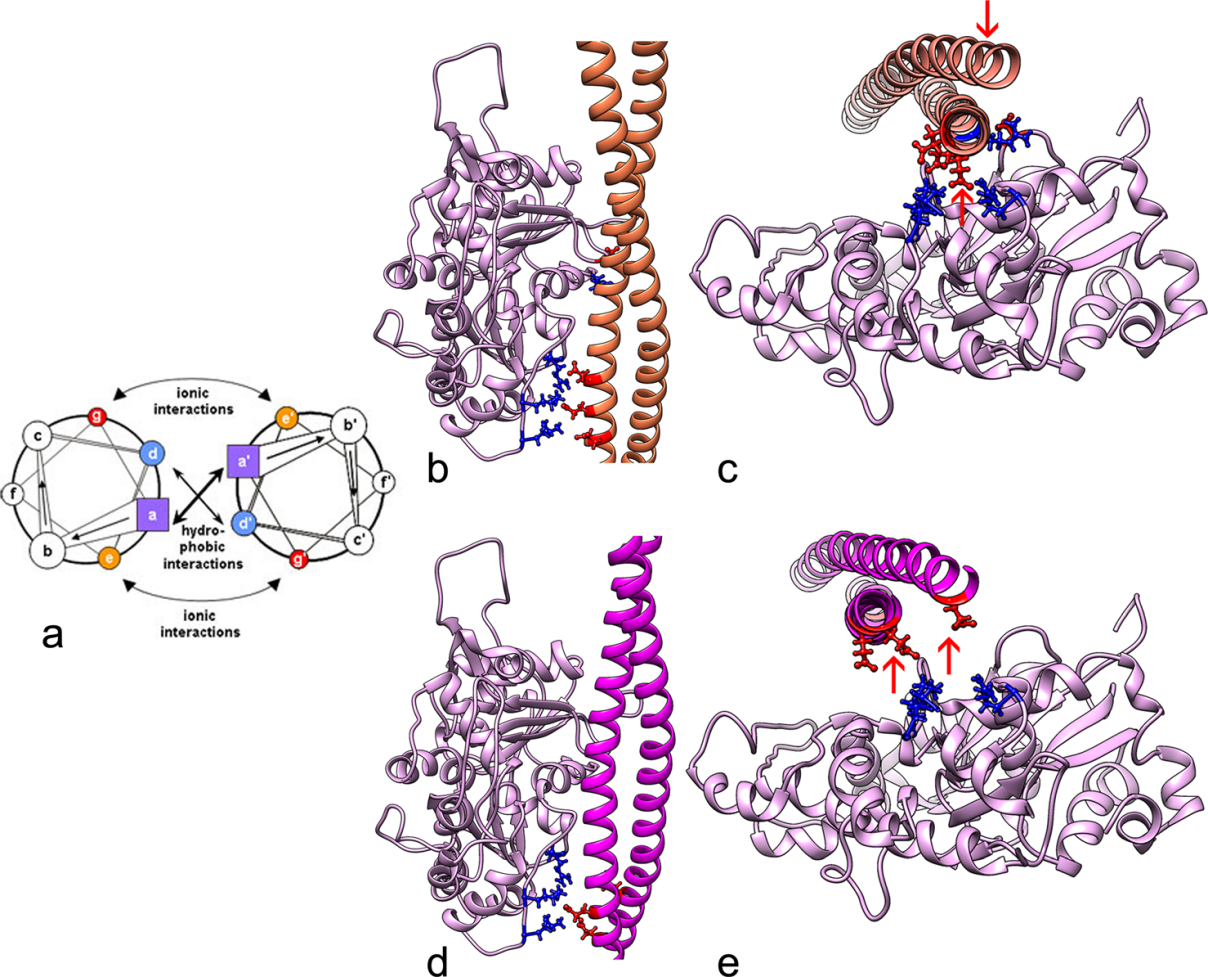
Coiled-coil orientation. (a) Helical wheel diagram of a dimeric parallel coiled coil; heptad positions are labeled *a* to *g* and *a*′ to *g*′, respectively, and positions *a, d, e,* and *g* are colored; Adapted from Hagemann *et al.* (2008). (b)–(e) Side chain interactions between oppositely charged amino acids of F-actin and one of the tropomyosin pseudo-repeats (in this case tropomyosin pseudo-repeat 5 (residues 165 to 205); *N.B*. comparable interactions with successive actin subunits occur for all tropomyosin pseudo-repeats (Orzechowski *et al.*, 2014)). (b) and (d) Side views of the actin-tropomyosin interaction surfaces ((b) model A and (d) model A′), (c) and (e) End-on views down the filament ((c) model A and (e) model A′). Side chains of residues involved in making electrostatic contacts are noted. In model A, these contacts originate from the narrow interface of tropomyosin which is pointed toward the surface of actin (arrows in (c)), while in model A′, interactions involve the wide tropomyosin interface lying parallel to the surface of actin (arrows in (e)). Note that in (b) and (c) tropomyosin residues 184, 181, and 177 in model A contact actin residues K326, K328, and R147 and tropomyosin residue R167 contacts actin D25. In the case of model A′, (d) and (e), residues E196, E194, and E192 of tropomyosin contact actin K326, K328, and R147; no contacts are made with actin D25 or R28.

As expected, the underlying amino acid sequence of tropomyosin defines the distinctive curvature of tropomyosin and its binding to F-actin (Brown *et al.*, 2001 and Brown and Cohen, 2005). In general, α-helical chains in coiled-coils are characterized by a seven-fold “heptapeptide” amino acid repeating pattern (labeled *a-g* in Figs. 3 and 4(a)). Hydrophobic “core” residues (*a* and *d*) are responsible for a hydrophobic “stripe” that holds a two-chained coiled-coil together, and, in tropomyosin, the core is bent into a super-helical shape by the patterning of alanine clusters among these residues (Brown *et al.*, 2001 and Brown and Cohen, 2005).

Cartoons of coiled-coils commonly diagram component heptapeptides in projection, viewed down their longitudinal axis (Fig. 4(a)) (Mason *et al.*, 2006 and Hagemann *et al.*, 2008), for example, schematically identifying hydrophobic *a* and *d* residues holding the two α-helices together. Polar and charged residues on the coiled-coil surface (*b, c, f*) face solvent (or actin or other proteins). Here, *f* residues lie furthest from the hydrophobic core and *b* and *c* sites more tangentially. In contrast, *e* and *g* residues likely form inter-chain salt bridges which additionally stabilize and align the coiled-coil structure (Parry, 1975 and McLachlan and Stewart, 1976). When instead coiled-coils are viewed face-on (as in Fig. 2), the two-chains show alternating wide and narrow interfaces as the α-helical chains wind around each other over each actin subunit. Thus, when illustrated in projection, as in Figure 4(a), the wide aspect of the coiled-coil shows *f* residues emerging outwards (and oriented in-plane). An orthogonal view in and out of the page would depict a projection of the narrow aspect of the coiled-coil now with a perpendicular orientation of *f* residues.

Figure 3 highlights different sets of charged residues on the tropomyosin surface that contact F-actin in the two alternative models of actin-tropomyosin discussed above and illustrated in Figure 2. Interestingly, the atomic model for the lowest energy minimum (Figs. 2(a) and 2(d)) largely exploits electrostatic contacts from tropomyosin's α-zone *f*-position residues and secondarily from *b*-position ones (Fig. 3). Here, the narrow interfaces of tropomyosin interact closely with successive actin subdomains 1 and 3 as tropomyosin wraps around F-actin (Figs. 4(b) and 4(c)). In contrast, the atomic model of F-actin-tropomyosin defined by the secondary energy minimum (Figs. 2(b) and 2(e)) appears to involve a smaller number of contacting residues (hence the shallower energy minimum). Because tropomyosin is shifted longitudinally by 24 Å, now the wide interface of tropomyosin lies closest to actin subdomains 1 and 3 with constituent side chains from two chains obliquely oriented to their targets (Figs. 4(d) and 4(e)). Here, mostly *g*-position β-zone residues interact with actin. Hence, tropomyosin is not only bent to the shape of actin, but component pseudo-repeats in each of the models are appropriately twisted to optimize actin-tropomyosin charge-charge sequence complementarity. The distinctive geometry that distinguishes these contacts in each of the models is retained over every tropomyosin pseudo-repeat and actin (Lehman *et al.*, 2014). Testing the effect of appropriate charge-reversal mutation of β-zone residues on the binding and assembly of thin filaments would be valuable.

### Mind the gap

Tropomyosin forms a continuous cable from one end of the fully assembled thin filament to the other. No gaps are observed in the cable which might expose filament segments containing actin free of tropomyosin. Indeed, gaps of any significant length would interfere with thin filament steric-regulation and its cooperativity. Nevertheless, nothing distinguishes one series of seven contiguous actin subunits from another along F-actin, and thus there is no reason *a priori* to expect tropomyosin to discriminate between different groups of successive actin subunits during filament assembly without leaving gaps. In fact, experimental evidence suggests that initial associations of muscle tropomyosin along filaments are random (Wegner, 1980 and Schmidt *et al.*, 2015) and that chain formation begins by tropomyosin growing out-of-register from nucleation sites, hence, resulting in gap formation during early stages of thin filament assembly (Schmidt *et al.*, 2015). Subsequently during filament maturation, gaps are eliminated; however, the mechanism of gap closure remains obscure, since microscopic resolution attained thus far has been insufficient to follow the respective process at a single molecule level (Schmidt *et al.*, 2015).

We propose that gaps shorter than seven actin subunits in length (into which one additional tropomyosin would not fit) are filled in during tropomyosin assembly on F-actin by the diffusion of either tropomyosin monomers or end-to-end linked tropomyosin oligomers “shuttling” over F-actin. If single molecules or short oligomeric chains can shuttle, i.e., translate longitudinally, over the actin surface, for example, between local energy minima, then end-to-end interactions will inevitably result to fill the gap and stabilize the resulting growing cable, as detailed below. Indeed, Holmes and Lehman (2008) and Schmidt *et al.* (2015) previously proposed that nascent tropomyosin cable formation would be followed by a stochastic mechanism in which gaps are filled between tropomyosin chains on F-actin as earlier modeled by Vilfan (2001). Nonetheless, unidirectional tropomyosin growth observed in non-muscle cell filaments may be facilitated by specific nucleation events, such as by formins which appear to promote non-muscle tropomyosin assembly beginning at filament barbed ends (Johnson *et al.*, 2014 and Gunning *et al.*, 2015). These studies and additional work by Hsiao *et al.* (2015) suggest that stable nucleation site(s) at one end of the actin filament will bias recruitment of tropomyosin monomers or nascent tropomyosin cables to that end. Such a process may also ensure that tropomyosin molecules in respective cables on both of actin's helical strands are in phase and will facilitate unidirectional tropomyosin polymerization. Whether or not nucleating-proteins participate in tropomyosin assembly on muscle thin filaments remains uncertain, and conversely, the extent to which tropomyosin promotes or inhibits the initiation of cellular actin filament polymerization and its elongation is not clear. In fact, tropomyosin present on filaments reconstituted from F-actin and smooth muscle tropomyosin, absent other actin-binding proteins, generates a 37.5 ± 1.8 nm periodicity characteristic of an in-register gap-free tropomyosin ensemble (Matsumura and Lin, 1982). Thus here, the precision of muscle filament assembly process appears to be governed by attributes inherent to F-actin and tropomyosin conformation alone.

The mechanism that we envision for the assembly of tropomyosin on the actin filament is illustrated schematically in Movie 1 (see supplementary material). Here, several tropomyosin molecules are shown binding and diffusing along and around the actin helix, shifting between binding minima described earlier that are dictated by their respective α- and β-band interactions. Sometimes individual tropomyosin molecules bind to actin targets, sometimes they dissociate, until eventually two molecules connect to each other on actin end-to-end. The cable can then elongate by a few tropomyosin molecules, as additional tropomyosins associate locally. The "breathing" of the newly formed, but short tropomyosin chain hovering over the actin filament, is caused by fluctuations in super-helical radius of the nascent cable largely directed away from the central axis of the F-actin. This can result in a partial helical unwinding of the short cable from F-actin. The “breathing” and unwinding can occur so long as the cable is still short, since each constituent tropomyosin binds so weakly to actin. Any further partial dissociation would also allow short cables to drift as if diffusing along the actin filament and then be enlisted by end-to-end bonding to other nascent tropomyosin chains to fill gaps in the cable. This process continues, followed by a helical re-tightening, ultimately forming a full-length tropomyosin cable that has no gaps.

The possibility of one-dimensional diffusion of tropomyosin over F-actin seems reasonable given estimates of the diffusion constant for tropomyosin as 2.2 × 10^−7^ cm^2^/s (Loong *et al.*, 2012) and the K_a_ for single tropomyosin molecules binding to F-actin (∼2 × 10^3^ M^−1^) (Wegner, 1980). It therefore follows that if single tropomyosin molecules have a dwell time on F-actin of 50–100 *μ*s, they then could diffuse roughly 40–70 nm over F-actin before dissociating. This distance would easily suffice for tropomyosin molecules to glide between adjacent energy minimum locations on F-actin and encounter potential binding partners.

The shuttling of tropomyosin over the surface of F-actin that we propose would necessarily involve large energy barriers if the nascent tropomyosin cable remained closely wrapped around helical F-actin (i.e., making close contacts with F-actin of the sort which yield the optimized energy landscapes). In order to perform the proposed shuttling, the nascent tropomyosin cable must helically unwind by a small amount, thus allowing for unhindered 1D-diffusion along the F-actin. This mechanism is fully consistent with the known weak binding affinity of tropomyosin for F-actin: we expect that the weak binding of tropomyosin allows a simultaneous partial unbinding of all the tropomyosin monomers within a short nascent cable (while still retaining all end-to-end connections between tropomyosins and tolerating the partial super-helical unwinding). In contrast, once the tropomyosin cable has extended sufficiently to spiral a few times around the actin helix, it is unlikely to be able to readily unwrap and dissociate from actin, provided that end-to-end connections are stable (Orzechowski *et al.*, 2014). If, alternatively, the tropomyosin end-to-end connections were weak, fully formed tropomyosin cables would typically dissociate from actin, contrary to observation. It follows that if disassembly can be excluded on these grounds, then there must be significant shuttling of short tropomyosin spirals along F-actin.

### Form-function relationships disfavor induced-fitting of tropomyosin on F-actin

The diffusion-based stochastic scheme that we propose for actin-tropomyosin binding differs fundamentally from ones more akin to induced-fitting mechanisms associated with enzyme-substrate interactions. In the case of the assembly of actin-tropomyosin, such an alternative process might rely on a marked conformational flexibility of tropomyosin, as has been implied by a number of investigators (e.g., see Whitby and Phillips, 2000; Singh *et al.*, 2003; Nitanai *et al.*, 2007; and Nevzorov and Levitsky, 2011). Here, the initial conformation of isolated tropomyosin would be expected to be relatively “uncommitted” to the shape of the F-actin helix (contrary to our observations). Tropomyosin would likely link transiently to targets on the actin surface and then flexibly fit to specific binding sites on actin subunits, driven by permissive energetic pathways. The susceptibility of tropomyosin to localized thermal unfolding and proteolysis is suggestive of the requisite conformational flexibility (Sumida *et al.*, 2008). Moreover, specific bends observed in some crystal structures of tropomyosin indicate some “undulation” in tropomyosin conformation (Whitby and Phillips, 2000 and Nitanai *et al.*, 2007). While provocative, none of these studies directly quantified tropomyosin's curvature variation (i.e., inherent flexibility), or whether or not local variance in tropomyosin curvature or flexibility can propagate along tropomyosin in a concerted manner (see Zheng *et al.*, 2013). Indeed, local vibration of adjacent or distant residues or their side-chains might offset each other or sum in complex and unpredictable ways, particularly after the protein links to actin.

As mentioned, effective tropomyosin cable formation on F-actin requires eliminating gaps formed during polymer assembly. Tropomyosin must relocate from initial binding sites on actin subunits in order to bind end-to-end. If conformational plasticity and induced-fitting are central to actin-tropomyosin interaction and filament assembly, then as binding events occur and are undone, the fitting would have to be repeated over and over until tropomyosin molecules find appropriate matches on F-actin in order to link end-to-end. Such a cyclic induced-fitting process seems complex and possibly energetically expensive, particularly if the cycling were to involve short tropomyosin oligomers. Instead, we propose a simpler and more parsimonious mechanism in which pre-shaped tropomyosin essentially floats over the actin surface, prejudiced by favorable but very weak associations, and then readily diffuses to meet partners in order to form a gap-free cable. Holmes and Lehman (2008) named the form-function relationship involved in tropomyosin binding to actin *Gestalt-binding*, which we find has compelling logic, considerable experimental support and now a testable framework based on sequence analysis and energy landscape measurements.
